# Tumor-derived Cav-1 promotes pre-metastatic niche formation and lung metastasis in breast cancer

**DOI:** 10.7150/thno.79250

**Published:** 2023-03-13

**Authors:** Yi Wang, Yuqiu Li, Junpei Zhong, Miao Li, Youjia Zhou, Qing Lin, Siwen Zong, Wenting Luo, Jiayang Wang, Keqin Wang, Jie Wang, Lixia Xiong

**Affiliations:** 1 Department of Pathophysiology, Medical College, Nanchang University, 461 Bayi Road, Nanchang 330006, China; 2 Queen Mary college, Nanchang University, Nanchang 330006, China; 3 Second Clinical Medical College, Nanchang University, Nanchang 330006, China; 4 First Clinical Medical College, Nanchang University, Nanchang 330006, China; 5 Key laboratory of functional and clinical translational medicine, Xiamen Medical College, Fujian province university, Xiamen 361023, China

**Keywords:** exosome, caveolin 1, pre-metastatic niche, breast cancer, lung metastasis

## Abstract

**Rationale:** Breast cancer (BC), as one of the most frequently diagnosed cancer, has a poor prognosis due to the development of distant metastasis. Among the BC metastatic sites, lung is one of the most common sites. Caveolin-1 (Cav-1) is a functional membrane protein that plays a vital role in tumor metastasis. Although studies have revealed that Cav-1 levels were elevated in patients with advanced cancer, whether Cav-1 affects BC lung metastasis by influencing the formation of pre-metastatic niche (PMN) through exosomes has not been explored.

**Methods:** Differential ultracentrifugation, transmission electron microscopy and nanoparticle tracking analysis were used to verify the presence of exosomes. Transwell assays were used to examine the biological effects of exosomes containing Cav-1. Both in vitro cell cultures and mammary tumor cell-induced mouse models were used to assess the lung metastasis. The regulatory mechanisms of PMN formation were revealed using western blot, flow cytometry, RT-qPCR, immunofluorescence assays, gene overexpression assays and RNA interference assays.

**Results:** Exosomes have critical functions in transporting Cav-1 between primary BC and metastatic organ microenvironments. Cav-1 in BC-derived exosomes can act as a signaling molecule to mediate intercellular communication and regulate the PMN before lung metastasis by regulating the expression of PMN marker genes and inflammatory chemokines in lung epithelial cells, promoting the secretion of tenascin-C (TnC) in lung fibroblasts to cause extracellular matrix (ECM) deposition, and inhibiting the PTEN/CCL2/VEGF-A signaling pathway in lung macrophages to facilitate their M2-type polarization and angiogenesis.

**Conclusion:** Our study investigated the mechanisms of lung PMN formation induced by Cav-1 in BC-derived exosomes. Our data may provide new directions for exploring the mechanisms and developing treatment strategies of BC lung metastasis.

## Introduction

According to global statistics of 2020, Breast cancer (BC) is the most common malignant tumor in women and the second leading cause of cancer-related deaths worldwide 1. Among breast cancer-related deaths, 90% are caused by cancer metastasis. Current studies have found that BC lung metastasis is one of the most common types of BC metastasis 2. However, the mechanism of its occurrence is unclear, and there are no effective metastasis markers and treatment methods in the clinic.

Tumor metastasis is a multi-stage process regulated by multiple factors 3. Researchers discovered that tumor metastasis to distant organs is influenced by interactions between tumor cells and the microenvironment of metastatic organs. Tumor cells form the pre-metastatic niche (PMN) by educating cells of distant metastatic organs, which is conducive to tumor cell colonization and growth, providing fertile soil for tumor cell metastasis 4. In lung metastasis, lung epithelial cells, the most common stromal cells, can be activated in a variety of ways to release cytokines and chemokines, resulting in abnormal activation of downstream signaling pathways and long-term chronic inflammatory responses 5. Moreover, lung fibroblasts and macrophages promote extracellular matrix (ECM) deposition and PMN formation 5, 6. Recent research has discovered that when lung fibroblasts receive signals from tumor cells, they can secrete tenascin-C (TnC), one of the important components of ECM, to form TNC-rich niches and initiate early ECM deposition 7. Multiple factors can activate lung macrophages, causing M2-type polarization, the release of chemokines to activate downstream pathways, and the maintenance of chronic inflammatory responses 6, 8. All of the above changes can promote the formation of PMN and facilitate tumor cells' colonization and metastasis. Therefore, studying the changes in the lung microenvironment and important molecules in the progression of BC is critical to understand and prevent the lung metastasis of BC.

However, during the formation of tumor PMN, tumor cells need certain carriers to interact with cells of metastatic organs. Exosomes, as tiny vesicles that carry biological signals, have been illustrated to transmit biological information and educate distant organs to form PMN in various tumors 9. Exosome is a kind of extracellular vesicles derived from multivesicular bodies (MVB), with a diameter of 30-150nm, which is widely present in various body fluids of the human body. It can mediate communication between cells and stroma by transporting its packaged cargo 10-12. At present, 32267 proteins, 639 lipids, 17005 mRNAs, and 2431 microRNAs have been found in exosomes from different tissues and cells 13. Exosomes secreted by tumor cells can have autocrine and paracrine effects in the local tumor microenvironment, such as arranging the primary tumor microenvironment, promoting angiogenesis, and reconstructing ECM and other biological effects. They can also exert endocrine effects through blood circulation to distant target organs, such as inducing PMN production in target organs to promote tumor metastasis 14. Besides, in lung and liver metastasis models of the tumor, recipient cells can recruit bone marrow-derived cells by recognizing tumor exosomes to promote inflammatory chemokine secretion and receptor expression, PMN marker gene expression, fibronectin synthesis, and angiogenesis 15, 16.

Caveolin-1(Cav-1) is a functional protein of caveolae, which has regulatory effects on molecules gathered in specific concave regions of the cell membrane. The transport of intracellular and extracellular substances, endocytosis of cells, and regulation of cellular signaling pathways are associated with Cav-1. Through its scaffolding region, it helps the transmission of intracellular information 17. More and more evidence has revealed that Cav-1 plays a vital role in different processes related to metastasis, such as epithelial-mesenchymal transition (EMT), angiogenesis, cell migration, ECM degradation, and sedimentation. Cav-1 is not only an intracellular protein that regulates cell function but also present in extracellular space and closely associated with cancer cell metastasis. As a secretion protein, Cav-1 can be detected in the serum of gastric cancer and advanced prostatic cancer patients, while the serum concentration of Cav-1 is significantly lower in normal people 18, 19. In prostate cancer, the secretion of Cav-1 by tumor cells leads to an increase in the non-adhesion growth ability of tumor cells 18. Besides, further studies have revealed that this secretory Cav-1 is present in tumor-derived exosomes 20. Through exosomes, tumor cells can be helped to mediate intercellular communication in specific microenvironmental pH conditions, thus promoting the exchange of specific molecules such as Cav-1, which lead to the phenotypic changes of recipient cells and enhance tumorigenicity 21. All of the above studies indicated that Cav-1 may be involved in regulating intercellular communication in BC with the help of tumor-derived exosomes.

At present, no relevant studies have been conducted on whether Cav-1 affects the formation of PMN through exosomes. By analyzing the cellular communication between primary and secondary tumor microenvironments, we delineate the specific roles and mechanisms of Cav-1 in lung metastasis of BC by focusing on the formation of PMN, which could increase the expression of PMN-related genes in lung epithelial cells, promote ECM deposition in lung fibroblasts and facilitate the M2 polarization of lung macrophages.

## Materials and Methods

### Cell culture

Human BC cell lines (MDA-MB-231 and MCF-7) and human lung fibroblasts HFL-1 and lung macrophages THP-1 were bought from Shanghai Cell Bank, Chinese Academy of Sciences. Cells were cultured in Dulbecco's Modified Eagle Medium (DMEM, Hyclone, USA) with 10% fetal bovine serum (FBS, BI, USA).

### Cell transfection

When cells had grown to about 70% of the bottom of the dish, plasmid were transfected respectively to the different groups of cells. Lipofectamine 3000 (lipo3000, Thermo Fisher, USA) and Opti-MEM serum-free medium (Gibco, USA) were used according to the instructions of transfection. After transfection and further culture for 6 hours, cells were changed into medium without exosome and serum and cultured for 48h for subsequent experiments. We set up 3 control groups, namely blank, siCON and pcCON and 2 experimental groups, i.e. the siCav-1 group for exosomes secreted by BC cells after the downregulation of Cav-1 (siCav-1) and the pcCav-1 group for exosomes secreted by BC cells after the overexpression of Cav-1 (pcCav-1).

### Exosome extraction

When cell density was 60%-80%, the supernatants of transfected cells were collected. Exosomes were extracted by sequential centrifugation steps. The collected supernatants were centrifuged successively at 300 g× for 10 minutes, 2000 g× for 10 minutes and 10000 g for 30 minutes. The supernatants were collected and put into an ultrafiltration tube and centrifuged at 5000 g× for 50 minutes. Finally, the supernatants were diluted with phosphate buffered saline (PBS).

### Exosomes identification

Exosome samples were diluted in PBS at 5:1000, so that 10-100 particles were obtained per image. Each group of samples was captured by NanoSight NS300 (NanoSight Nanoparticle Tracking Analysis 2.3 Nanoparticle Tracking and Analysis Release Version Build 0033; Worcestershire, UK) and a nanoparticle tracking analyzer, and provided 3 videos lasting for 30 seconds. Then the resulting videos were analyzed to measure the average and model particle sizes (particle size distribution) and particle number (concentration) of exosomes in each group. Besides, we also used Philips Tecnai 12 BioTwin transmission electron microscope (Pontificia Universidad Cat´olica de Chile, Santiago, Chile) operating at 80 kV to acquire the images of exosomes for identification.

### Transwell assays

Based on the instructions, transwell assays were carried out in Boyden Chambers (Transwell Costar) and invasion assays were performed in these chambers lined with Matrigel. 1x10^5^ cells previously treated with the supernatant of different groups of transfected cells during 24 hours were resuspended in serum-free medium and added to the top of the upper chamber containing 200ul serum-free medium. 24 hours later, the medium in the upper and lower chamber were discarded and washed up transwell chambers 3 times by PBS. Cells that migrated in the lower chamber were stained for 20 minutes with 0.1% crystal violet after using 4% paraformaldehyde for fixation. Finally, five photos were acquired. The cells counts were averaged.

### Immunofluorescence

After using the supernatant of different groups of transfected MDA-MB-231 to further culture lung fibroblasts, cells were fixed with 2 ml 4% paraformaldehyde (acetone: methyl alcohol = 1:1) at room temperature for 30 minutes. Then, 2 ml 5% BSA and PBS containing 0.3% Triton-100 were added for 2 h. Diluted primary antibodies (anti-TNC) (Affinity, USA) were added and cells were subsequently incubated at 4 ℃ overnight. Next, immunofluorescent secondary antibodies was used to continue incubating cells at 37 ℃ for 2 h in dark condition. Finally, added 5 µL of 4'6-diamidino-2-phenylin-dole (DAPI) agent was added to visualize cells under the fluorescence microscope (Leica Microsystems, Germany).

### Western blot analysis

Proteins in cells were extracted using RIPA lysis buffer (Beijing Solarbio Science &amp; Technology Co. Ltd., Beijing, China). 8ul denatured sample proteins and 4 µL markers were loaded into 12% Sodium Dodecyl Sulfate (SDS) gel and separated by SDS-polyacrylamide gel electrophoresis. After that, we used electrotransfection to transfer the proteins to PVDF membranes (Millipore Corp, billerica, MA, USA) at 200mA for 1h. Then, Transferred PVDF membrane was blocked in incubation liquid (TBST+dried skim milk) for more than 2 h. Next, we incubated the PVDF membrane with primary antibody (anti-TnC, anti-fibronectin, anti-nidogen, anti-emilin, anti-VEGF-A, anti-VEGF-B, Abcam, USA; anti-CCL2, Proteintech, China) at 4 °C overnight. After washing with TBSP and the membrane was incubated with secondary antibodies (IgG-HPR of goat anti-rabbit and goat anti-mouse, Beijing Zhongshan Jinqiao, China) for 2 h at room temperature. Finally, we scanned the blots using a gel imaging system and used software to calculate the gray value.

### RNA extraction and quantitative RT-qPCR

The extraction of RNA was reverse transcribed into cDNA by EasyScript®RT/RI Enzyme Mix before RT-qPCR. RT-qPCR was carried out using 2×TransStart® Tip Green qPCR Supermix and a StepOne RT-qPCR machine. To normalize the expression of target genes, GAPDH was used as an endogenous control gene. The thermocycling program is composed of holding at 94 °C for 30 s, followed by 40 cycles of 5 s at 94°C and 30 s at 62 °C, then 15 s at 95 °C, 1 min at 60 °C and 15 s at 95 °C. Then, collected melting curve data to verify RT-qPCR specificity and the absence of primer dimers. Relative quantity of target mRNA was calculated and presented in 2^-ΔΔCT^ method. The RT-qPCR primers are shown in the supplement material.

### Construction of mouse model

The skin of the right chest wall of mice was cut about 1.0 cm, exposing the second pair of mammary fat pads, into which 0.2 mL of prepared cells suspension was injected to construct the orthotopic BC model of mouse. Then, the incision was sutured. To educate the mouse model, exosomes and blank group (equal volume PBS) were injected into the different groups of mice through the tail vein, once every other day for one month.

### H&E staining and Immunohistochemistry

After exosome education in one mouth, the lung tissues were removed to observe the lung metastatic nodules of breast cancer. For HE staining, after paraffin sections were dewaxed and hydrated, they were stained with hematoxylin for 5-10 min and with eosin for 10 min. Immunohistochemistry staining was followed by the protocol of the Immunohistochemistry Kit (Sango Biotech, Shanghai, China).

### Flow cytometry

For determining the polarization of lung macrophages Thp-1, cells were incubated with allophycocyanin-conjugated human monoclonal CD68 antibody (Ab) (BD Biosciences, San Jose, CA) and phycoerythrin-conjugated human monoclonal CD206 Ab (BD Biosciences) for 30 min then subjected to cytometry analysis (FACS Calibur, BD Biosciences).

### Statistical analysis

All the data were represented as mean± standard deviation (‾x± SD). GraphPad Prism 9 software was used to make charts and SPSS17.0 software was used to analyze experimental data. The differences between two groups were analyzed by the student t-test, while One-way ANOVA method was carried out to analyze differences among 3 or more groups. p < 0.05 was defined as statistically significant. p < 0.01 was determined as highly statistically significant. p < 0.001 was considered as remarkably statistically significant. NS indicates no statistically significant difference between data.

## Results

### Cav-1 can be transported by BC-derived exosomes

**Figure 1:**
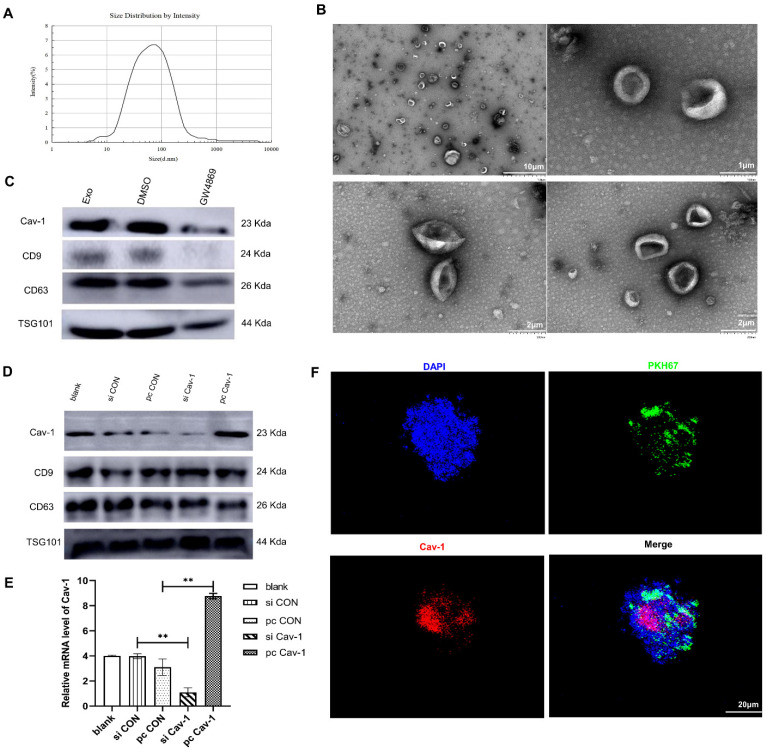
** BC cells release exosomes containing Cav-1. (A)** The results of nanoparticle tracking analyzer showed exosomes diameter. **(B).** Morphology of exosomes was showed using different magnifications by transmission electron microscopy. The scale of the upper left figure, the upper right figure and lower figure is 10μm, 1 μm and 2 μm.** (C).** Western Blot revealed the expression of CD63, CD9, TSG101 and Cav-1 in the supernatant of BC cells. CD63, CD9 and TSG are exosome marker proteins. GW4689 is an exosome inhibitor. **(D).** The expression level of Cav-1 protein in extracted exosomes from different groups was revealed by Western Blot. CD63, CD9 and TSG101 are exosome marker proteins. **(E).** RT-qPCR showed the mRNA level of Cav-1 in extracted exosomes from different groups. Data ware shown as mean ± standard deviation and assessed with Two-way ANOVA test. **(F).** Confocal microscopy images showed the transition of Cav-1 from exosomes into lung epithelial cells. Cav-1 was labeled in red, exosomes from MDA-MB-231 cells were labeled by PKH67 (green) and cell nuclei were stained with DAPI (blue). Scale bar=20µm. Data ware shown as mean ± standard deviation and assessed with One-way ANOVA test. (n = 3) (* p < 0.05; ** p < 0.01; *** p < 0.001)

Exosomes mediate cellular communication via transferring various nucleic acids and protein in different physiological and pathological situations. To detect whether exosomes can be used as vectors to transport Cav-1, firstly, we used transmission electron microscope and nanoparticle tracking analysis to identify BC-derived exosomes. As is shown in the **Figure 1A**, the exosome diameters were ranged from 30 nm to 150 nm, with the averaged diameter being 100 nm. The morphology of exosomes was mostly round and the edge was clear under the transmission electron microscope **(Figure 1B)**. In addition, the results of western blot revealed the expression of exosome specific marker proteins CD63, CD9, TSG101 (tumor susceptibility gene) in the supernatant of MDA-MB-231 cells, which further confirmed the presence of exosomes in BC cell supernatant. To elucidate the presence of Cav-1 in exosomes, MDA-MB-231 cell models without exosomes were constructed using exosome inhibitors GW4689. Results showed that the Cav-1 expression level in MDA-MB-231 cell culture media containing exosomes is higher than the cell mediums without exosomes **(Figure 1C)**. After that, we also collected the supernatants of MDA-MB231 cells, extracted exosomes and detected the expression of Cav-1 in exosomes, which convinced the vector function of exosomes. In our experiment, we used plasmids to further increase or decrease Cav-1 expression in pcCav-1 group and siCav-1 group respectively to explore the effects of different Cav-1 expression levels in BC cells. **Figure 1D** has showed the increased expression level of Cav-1 in pcCav-1 group and decreased expression of Cav-1 in siCav-1 group in exosomes. CD63, CD9 and TSG101 are exosome marker proteins. The different expression levels of Cav-1 in the extracted exosomes were also revealed by RT-qPCR **(Figure 1E)**. Collectively, we concluded that BC cells can transport Cav-1 by exosomes. Furthermore, we also verified the transportation of Cav-1 from BC-derived exosomes to lung epithelial cells via labeling Cav-1 in exosomes. Confocal imaging showed the presence of Cav-1 in the cytoplasm of lung epithelial cells, indicating that BC-derived Cav-1 could be delivered into lung epithelial cells from exosomes **(Figure 1F).** These results laid the foundation for further exploration of the effects and mechanisms of Cav-1 in BC-derived exosomes on the formation of lung PMN during BC metastasis.

### Cav-1 in BC-derived exosomes promotes the BC lung metastasis

**Figure 2:**
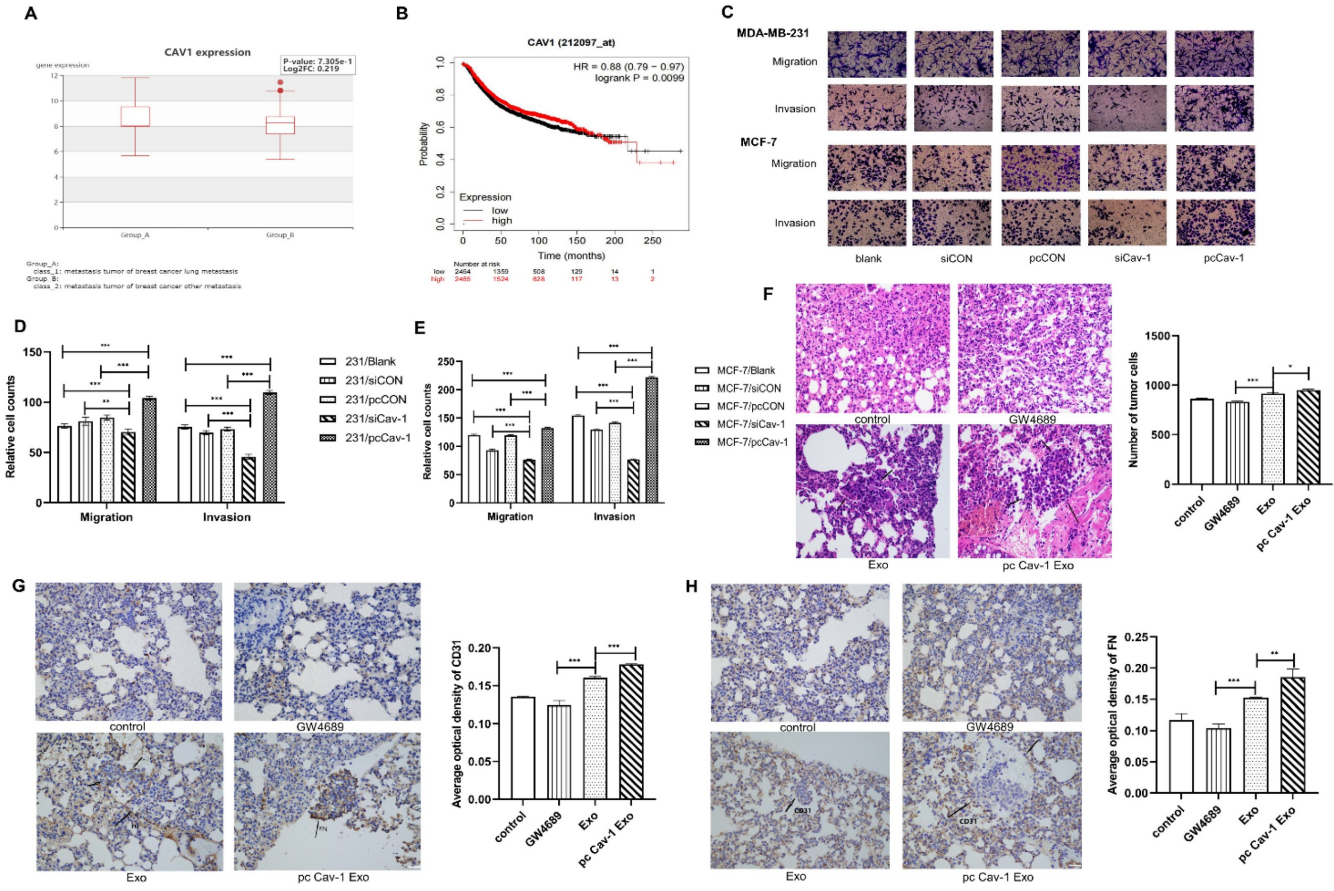
** Cav-1 in BC-derived exosomes promotes BC lung metastasis. (**A). BC lung metastatic patients have higher expression of Cav-1 than BC patients with other metastases. (B). Breast cancer patients with high expression of Cav-1 might have a poorer prognosis. (C, D, E). Transwell assays detected the invasion and migration of MDA-MB-231 cells and MCF-7 cells. Bar=100 µm (magnification: 200×)** (F).** H&E staining revealed the tumor cells in the lung tissue. Bar=100 µm (magnification: 200×).** (G).** Immunohistochemistry of FN in the lung tissues.** (H).** Immunohistochemistry of vascular endothelial cell marker CD31. Bar=100 µm (magnification: 200×). Data ware shown as mean ± standard deviation and assessed with One-way ANOVA test. (n = 3) (* p < 0.05; ** p < 0.01; *** p < 0.001)

Since we have confirmed the existence of Cav-1 in BC-derived exosomes, next, we explored the effects of Cav-1 in BC-derived exosomes on BC lung metastasis tissues. The bioinformatics data showed that BC lung metastatic patients have higher expression of Cav-1 than BC patients with other metastases **(Figure 2A)**. Besides, the Kaplan-Meier analysis suggested that patients with a high expression of Cav-1 may have a worse prognosis, which indicates the significance of Cav-1 in BC lung metastasis **(Figure 2B)**. To further investigate the effects of BC-derived exosomes containing Cav-1 on the invasion and migration ability of MDA-MB-231 cells and MCF-7 cells, transwell invasion and migration assays were used to. In the transwell assays, only cells with the ability to migrate or invade through the membrane or Matrigel were observed. There were decreased cell numbers in the siCav-1 group and increased cell numbers in the pcCav-1 group compared with control groups **(Figure 2C-E)**. This indicated that decreased Cav-1 in BC-derived exosomes reduced the migration and invasion ability of invasive MDA-MB-231 cells and non-invasive MCF-7 cells, while increased Cav-1 promoted the migration and invasion ability of these cells. Above observations suggested that Cav-1 in BC-derived exosomes could promote the invasion and migration ability of BC cells.

In addition to the cellular levels, we next explored the Cav-1 effects at animal levels. Mice models of orthotopic breast tumors were constructed and modified by injecting exosomes and exosomes with increased Cav-1 through tail veins to educate the lung microenvironment and form a PMN, meanwhile the PBS and exosome inhibitors were injected in control and compared groups, respectively. We evaluated the lung metastases through HE staining and immunohistochemistry on paraffin sections. In the HE staining, compared with the control group and exosome group, pcCav-1 group that had increased Cav-1 in the exosomes significantly enhanced the formation of lung metastases. Microscopically, the neoplastic cells with marked hyperchromatic nuclei and pleomorphism, even some tumor thrombi were observed. Besides, the decreased lung metastases in the exosome inhibitor GW4869 group also confirmed the Cav-1 effects in promoting lung metastasis **(Figure 2F)**. Moreover, immunohistochemical results of fibronectin (FN) showed that the expression level of FN in the pcCav-1 group was significantly higher than the groups treated with exosome alone **(Figure 2G)**. Immunohistochemical of vascular endothelial cell marker CD31 also showed strongly increased expression in the high expression level of Cav-1 exosome group, slightly increased expression in the exosome group and lower expression in the exosome inhibitor group** (Figure 2H)**. All of the above data confirmed that Cav-1 in BC-derived exosomes could promote fibronectin synthesis and angiogenesis in metastatic lung tissues of BC, and thereby contribute to BC lung metastasis.

### Cav-1 in BC-derived exosomes promotes ECM deposition in lung fibroblasts to contribute to the PMN formation

**Figure 3:**
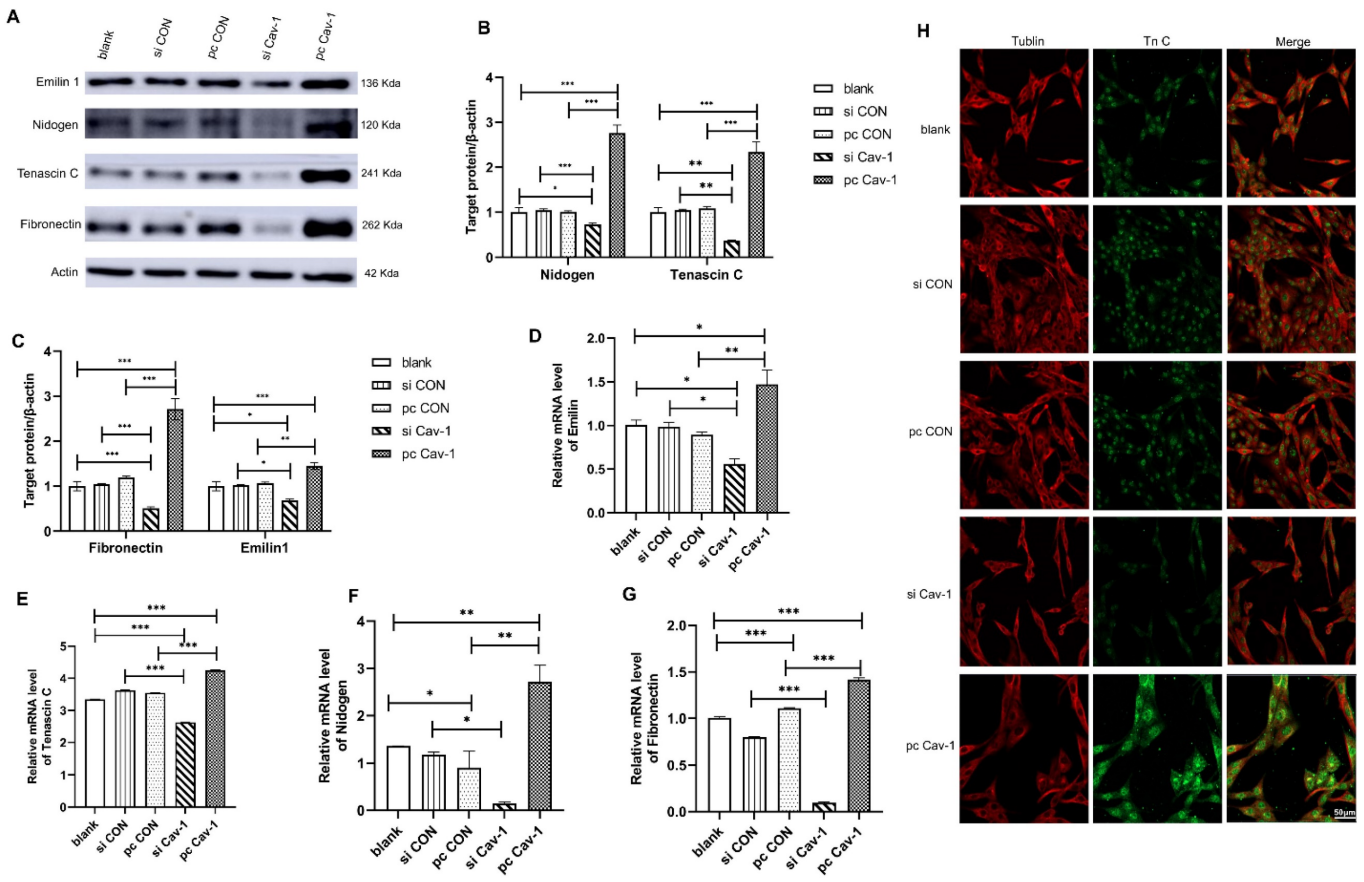
** Cav-1 in BC-derived exosomes promotes ECM deposition. (A, B, C).** Western blot detected the expression levels of ECM components emilin1, nidogen, TnC and FN in lung fibroblasts. **(D, E, F, G).** RT-qPCR revealed the mRNA expression level of ECM components emilin1, nidogen, TnC and FN in lung fibroblasts. **(H).** Immunofluorescent showed the ECM deposition in lung fibroblasts by evaluating the expression of TnC protein. Bar=50 µm (magnification: 400×). Data ware shown as mean ± standard deviation and assessed with One-way ANOVA test. (n = 3) (* p < 0.05; ** p < 0.01; *** p < 0.001)

Although we have illustrated that BC cells may transport Cav-1 from the primary tumor microenvironment to lung tissues through exosomes as carriers, educate lung tissues to form PMN and promote the occurrence of BC lung metastasis, the specific molecular mechanisms of lung PMN formation induced by BC-derived exosomes were still unclear. Therefore, to further verify the mechanism of PMN formation, we firstly focused on the ECM deposition in lung fibroblasts HFL1. After 48 h of cell transfection that increased Cav-1 or decreased Cav-1 in different groups of MDA-MB231 cells, the supernatant of the transfected cells was collected. Then the collected supernatants that contained each group of MDA-MB-231 cell-derived exosomes were used, respectively, to further culture lung fibroblasts HFL1. The expression level of ECM component proteins emilin1, nidogen, TnC and FN were detected by Western blot. The four ECM related proteins were highly expressed in pcCav-1 group but low expressed in siCav-1 group compared with control groups, which indicated that increased Cav-1 in BC-derived exosome promoted ECM deposition and decreased Cav-1 reduced ECM deposition **(Figure 3A-C)**. Consistent with this, the similar trend was demonstrated by RT-qPCR **(Figure 3D-G)**. Furthermore, the immunofluorescence assays of TnC, one of the ECM components proteins, revealed that the expression of TnC was decreased in siCav-1 group and increased in pcCav-1 group **(Figure 3H)**. Therefore, Cav-1 in BC-derived exosomes can promote lung fibroblasts to synthesize TnC and other proteins to facilitate the ECM deposition, as one of the mechanisms of lung PMN formation, which promotes BC lung metastasis.

### Cav-1 in BC-derived exosomes promotes the expression of PMN related genes in lung epithelial cells and M2 polarization in lung macrophages

**Figure 4:**
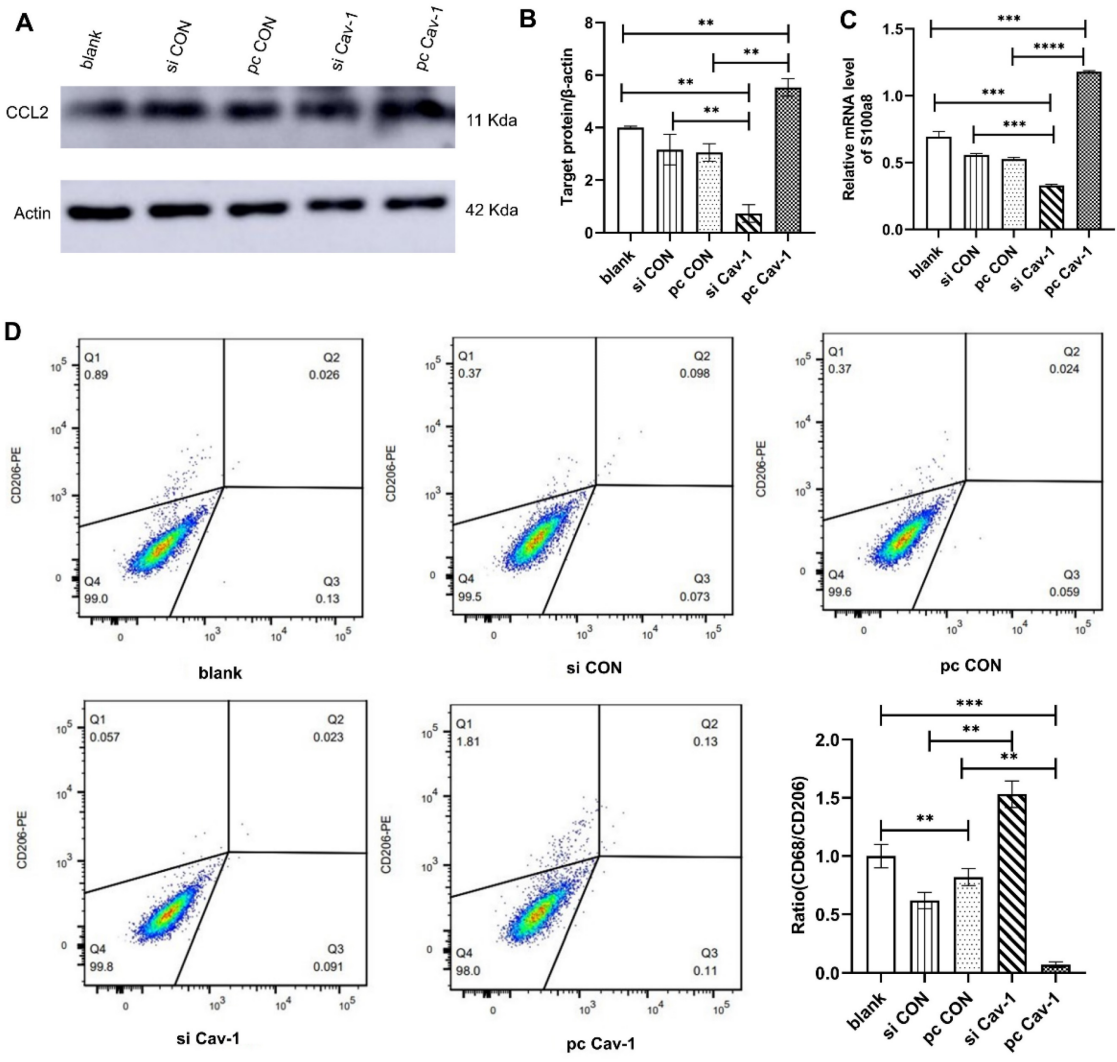
** The effects of BC-derived exosomes containing Cav-1 in lung epithelial cells and macrophages. (A, B).** Western blot showed the increased expression levels of CCL2 in pcCav-1 group in lung epithelial cells. **(C).** The mRNA expression level of s100a8 was increased in pcCav-1 group revealed by RT-qPCR in lung epithelial cells. **(D).** The analysis of flow cytometry illustrated the decreased expression of CD68/CD206 in the pcCav-1 group and increased expression in the siCav-1 group. Data ware shown as mean ± standard deviation and assessed with One-way ANOVA test. (n = 3) (* p < 0.05; ** p < 0.01; *** p < 0.001)

Similar methods were adopted to investigate the effects of Cav-1 in BC-derived exosomes in lung epithelial cells and macrophages. After extracting MDA-MB-231 cells-derived exosomes from different groups and culturing them with lung epithelial cells Beas2B, the expression level of PMN marker gene CCL2 was increased in the pcCav-1 group that had high expression level of Cav-1 and decreased in the siCav-1 group that had low Cav-1 **(Figure 4A-B)**. The results of RT-qPCR revealed the mRNA expression level of another PMN marker gene, s100a8, was increased with the high expression level of Cav-1 and decreased with the low expression level of Cav-1 **(Figure 4C)**. To explore the effects of Cav-1 in BC-derived exosomes in lung macrophages, flow cytometry analysis was conducted. CD206 is the surface maker of M1 macrophages while CD68 is the M2 macrophage maker. As is shown, the expression level of CD206 was decreased and the ratio of CD68 to CD206 was increased after the macrophages THP-1 were challenged using BC-derived exosomes containing decreased Cav-1. Opposite results were observed in the pcCav-1 group had increased Cav-1 expression level **(Figure 4D)**. The above results of flow cytometry indicated that Cav-1 in BC-derived exosomes could promote polarization of lung macrophages, which plays an important role in the lung PMN formation. Collectively, these data suggested that Cav-1 in BC-derived exosomes could promote PMN formation by stimulating PMN related genes in lung epithelial cells and facilitating M2 polarization of lung macrophages.

### Cav-1 in BC-derived exosomes inhibits the PTEN/CCL2/VEGF-A signaling pathway in lung macrophages to facilitate the PMN formation

**Figure 5:**
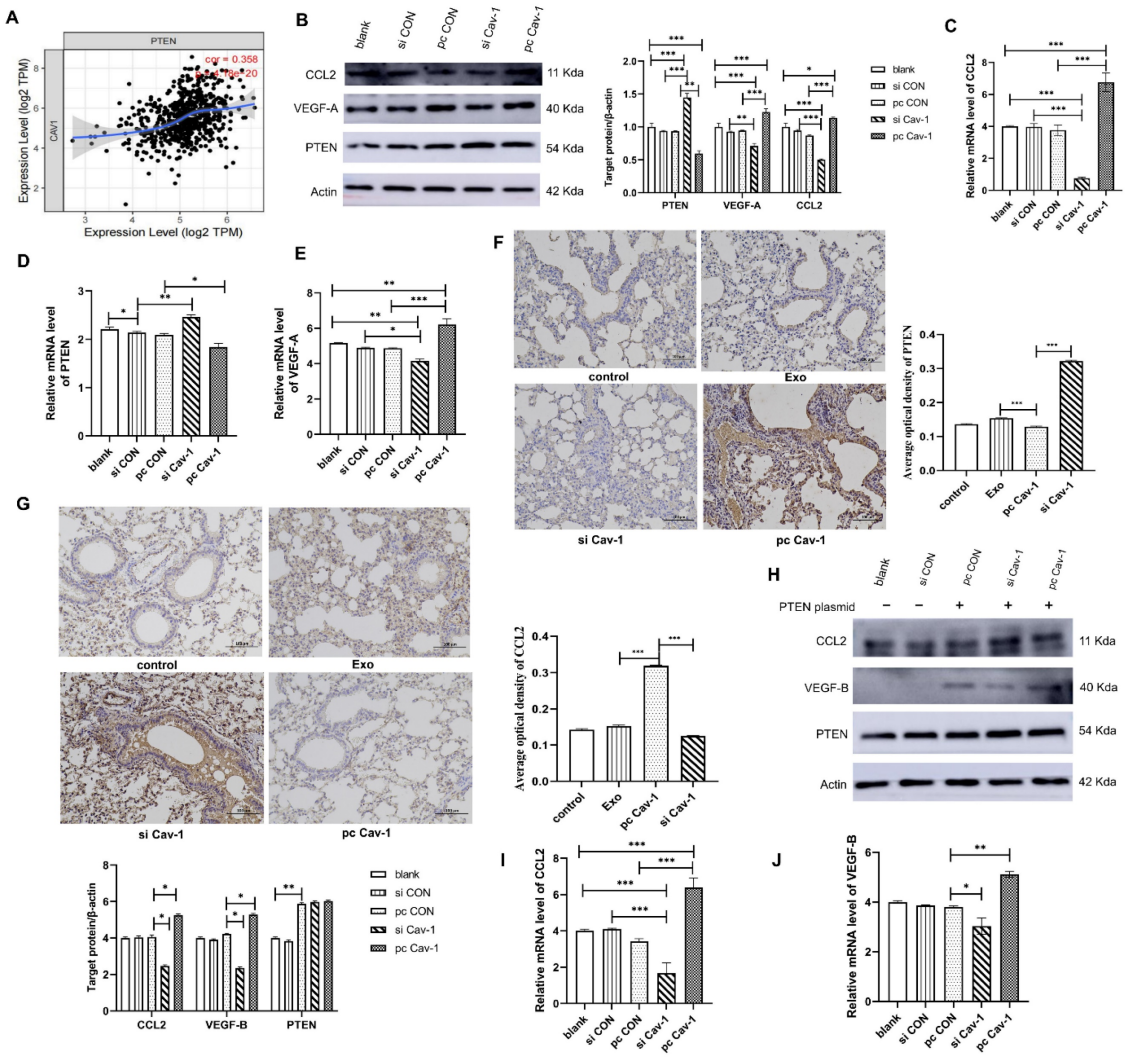
** Cav-1 in BC-derived exosomes inhibits the PTEN/CCL2/VEGF-A signaling pathway in lung macrophages. (A).** Statistical analysis showed that PTEN and Cav-1 have a correlation relationship.** (B).** Western blot analyzed the expression levels of PTEN, CCL2 and VEGF-A protein in lung macrophage THP-1.** (C, D, E).** The mRNA expression level of PTEN, CCL2 and VEGF-A in lung macrophages. **(F).** Immunohistochemistry of PTEN. Bar=100 µm (magnification: 200×). **(G).** Immunohistochemistry of CCL2. Bar=100 µm (magnification: 200×). **(H).** Western Blot analyzed the expression levels of CCL2 and VEGF-B protein in overexpressed PTEN lung macrophage models. **(I, J).** The mRNA expression level of CCL2 and VEGF-B in lung macrophages with overexpressed PTEN. Data ware shown as mean ± standard deviation and assessed with One-way ANOVA test. (n = 3) (* p < 0.05; ** p < 0.01; *** p < 0.001)

At this point, we have found that Cav-1 in BC-derived exosomes promoted the M2 polarization of macrophages. Next, we explored the specific signaling pathway involved in the process. Statistical analysis showed that PTEN and Cav-1 have a correlation relationship** (Figure 5A).** After stimulating lung macrophages THP-1 using MDA-MB-231 cell-derived exosomes, we performed western blot to detect the expression levels of PTEN, CCL2 and VEGF-A in the lung macrophages lysate. In the pcCav-1 group, the expression of CCL2 and VEGF-A increased and that of PTEN decreased, while the opposite result was observed in the siCav-1 group **(Figure 5B)**. The results of RT-qPCR also showed the similar trends **(Figure 5C-E)**. Furthermore, the in vivo experiments also revealed the increased expression of PTEN and decreased expression of CCL2 in pcCav-1 group **(Figure 5F-G)**. Altogether, the above data indicated that the high expression level of Cav-1 increased the expression of CCL2 and VEGF-A and decreased the expression of PTEN. To further identify the PTEN/CCL2/VEGF-A signaling pathway, we overexpressed PTEN in lung macrophages using pcDNA transfection and added the extracted BC-derived exosomes. After culturing for 24 h, we used western blot and RT-qPCR to measure the expression levels of CCL2 and VEGF-B (Vascular endothelial growth factor B). The expression of VEGF-B indicated macrophages changed into the M2 phenotype while VEGF-A suggested macrophages stayed in the M1 phenotype. Results have shown that the expression levels of CCL2 and VEGF-B in lung macrophages reduced after the treatment of BC-derived exosomes containing decreased Cav-1, while the expression level of CCL2 and VEGF-B increased in the high expression level of Cav-1 group **(Figure 5H-J)**. The results confirmed the influence of Cav-1 in BC-derived exosomes on the PTEN/CCL2/VEGF-A signaling pathway in lung macrophages. Since one literature has revealed that PTEN could decrease the expression of CCL2 and VEGF-A to inhibit macrophage polarization from innate macrophages to M2 phenotypes in the tumor microenvironment 7, combined with our results, we conclude that exosomes containing Cav-1 could inhibit the PTEN/CCL2/VEGF-A signaling pathway to promote the M2 polarization and angiogenesis of lung macrophages, which may play an important role in the PMN formation and BC lung metastasis.

### Cav-1 in BC-derived exosomes transports TnC to lung fibroblasts to facilitate the PMN formation

**Figure 6:**
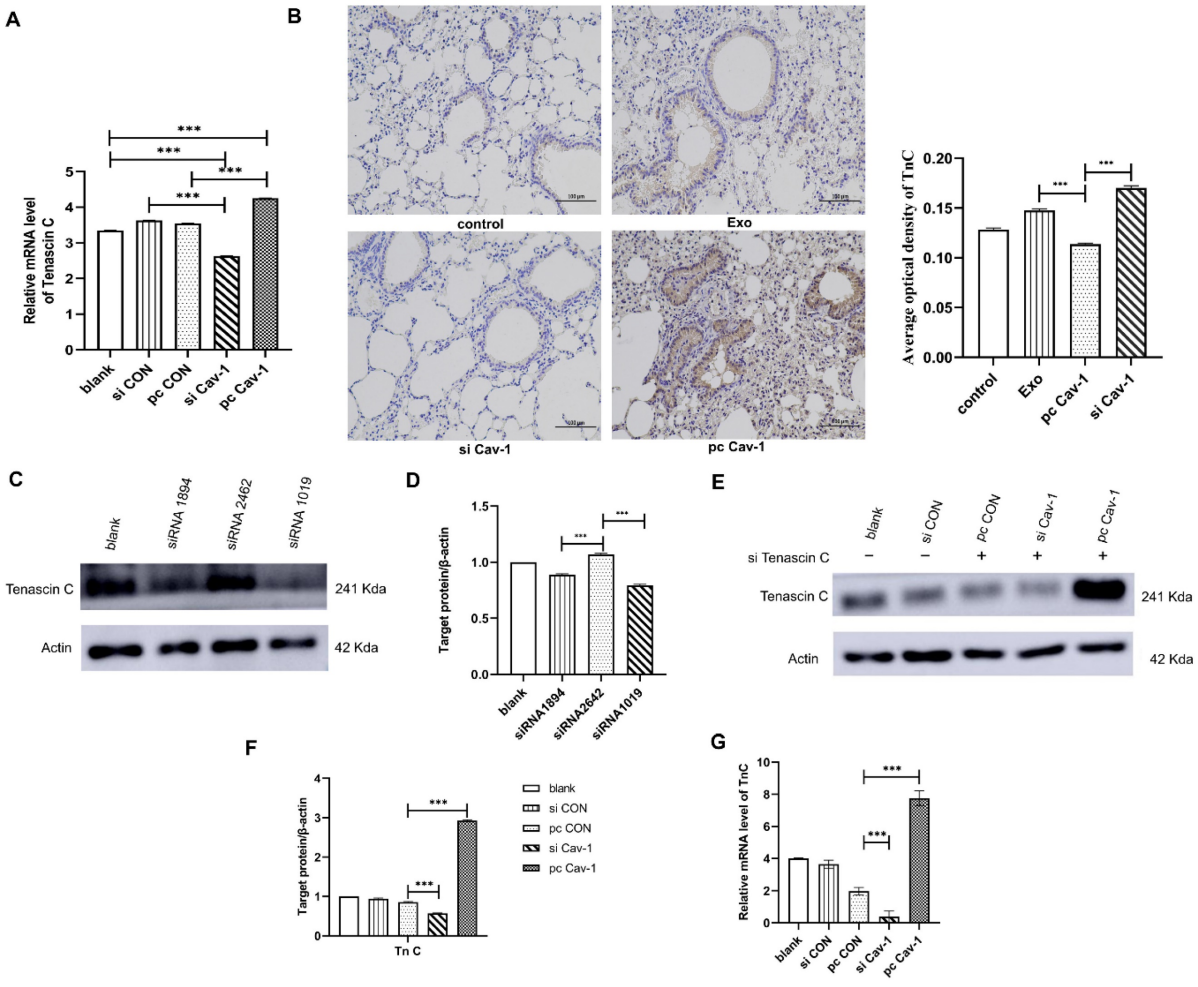
** Cav-1 in BC-derived exosomes transports TnC to lung fibroblasts. (A).** RT-qPCR revealed that TnC was decreased in BC-derived exosomes that have increased Cav-1 and increased in BC-derived exosomes with decreased Cav-1. **(B).** Immunohistochemistry of TnC. Bar=100 µm (magnification: 200×). **(C, D).** Western blot was used to detect the expression of TnC after transient transfection of different siRNA fragments in HFL1 cells. **(E, F).** Western blot showed the expression levels of TnC in lung fibroblasts HFL1 with knockdown TnC gene. The TnC genes in the pcCON, pcCav-1 and siCav-1 groups were knockdown. There were increased expression level in the pcCav-1 group and decreased level in the siCav-1group, which indicated that increased Cav-1 saved the expression of TnC and prevent it from decreasing, while in the decreased Cav-1 group, the expression of TnC was not saved and then decreased. **(G)** Consistent results were showed by RT-qPCR. Data ware shown as mean ± standard deviation and assessed with One-way ANOVA test. (n = 3) (* p < 0.05; ** p < 0.01; *** p < 0.001)

Since we have demonstrated that Cav-1 in BC-derived exosomes could promote ECM deposition in lung fibroblasts, to explore the underlying mechanisms, western blot and RT-qPCR were conducted to detect the expression level of TnC in lung fibroblasts. Results have showed that the expression level of TnC was decreased in BC-derived exosomes containing siCav-1 and increased in BC-derived exosomes containing pcCav-1 **(Figure 3A-B, 6A)**. Besides, the in vivo experiments also revealed the decreased expression of TnC in siCav-1 and increased expression in pcCav-1** (Figure 6B)**. To further explore the signaling pathway, we tried to knockdown the TnC gene in the lung fibroblasts HFL1. The results of western blot indicated the obvious TnC knockdown effects of siRNA1019 compared to siRNA1894 and siRNA2462 in HFL-1 cells **(Figure 6C-D)**. Therefore, we used siRNA1019 to knockdown the TnC gene in the lung fibroblasts HFL1. After adding the exosomes derived from different groups of BC cells to the lung fibroblasts HFL1 with TnC gene knockdown and culturing the fibroblasts, western blot was used to determine the expression level of TnC. The expression level of TnC in HFL1 cells was reduced after the treatment with exosomes containing decreased Cav-1 and increased after treating with exosomes containing high expression level of Cav-1, which suggested that increased Cav-1 expression saved the knockdown effects of TnC and prevent it from decreasing **(Figure 6E-F)**. RT-qPCR also showed the similar trend **(Figure 6G)**. All of the results suggested that Cav-1 in BC-derived exosomes could transport TnC to the lung stromal cells, which facilitated the ECM deposition in lung fibroblasts and served as a signaling molecule, promoting PMN formation in BC lung metastasis.

## Discussion

Although the BC death rates have continued to decrease in older women since 2007, the 5-year relative survival rates for BC patients whose cancer has spread to distant parts of the body are only 29%, which indicates the significance of studying the mechanisms of BC metastasis 22. As a key factor in facilitating tumor metastasis, PMN has attracted more and more attention. Studies have shown that exosomes could mediate communication between tumor cells by transferring their contents, such as proteins, mRNAs and/ or DNA fragments into the recipient cells to promote the formation of PMN 23.

Cav-1 plays a vital role in regulating various signaling pathways related to cell growth and migration 24. Cav-1 expressed by cancer-associated fibroblasts (CAF) is enriched in the stroma of many human cancer types such as breast, colorectal, kidney and metastatic melanomas 25. However, the role of Cav-1 in cancer metastasis is complex and controversial. In the early stages of cancer progression, the expression of Cav-1 is reduced and may has a primarily anticancer role; in the late stage, Cav-1 expression is positively correlated with tumor progression, multidrug resistance and metastasis 26, 27. The role of Cav-1 in cancer progression may vary depending on the type of cancer. In prostate stroma, low expression of Cav-1 promotes tumor progression, while in the CAF of BC, Cav-1 expression is related to a poor prognosis 25, 28. Although many studies concentrated on the function of Cav-1 as an intracellular protein, only a few studies have indicated that Cav-1 may be transported by exosomes and promote tumor metastasis by helping PMN formation.

In our study, by using exosome inhibitors, we found that Cav-1 expression levels were reduced in cancer cell cultures without exosomes, which indicated that exosomes can be used as vectors to transport Cav-1. Besides, to determine the specific effects of BC-derived exosomes in the tumor progression process, transwell experiment was conducted. The increased migration and invasion ability of invasive MDA-MB-231 cells and noninvasive MCF-7 cells at the high expression level of the Cav-1 group suggested that Cav-1 in tumor-derived exosomes could promote BC metastasis. To further confirm the effects of Cav-1 in promoting tumor metastasis, in vitro experiments by constructing in situ breast tumor mice models and injecting exosomes to educate the lung microenvironment were also conducted. After one month, HE staining of the lung tissues revealed the presence of obvious cancer cells and tumor thrombi in the increased Cav-1 group, which proved the contribution of Cav-1 in the formation of BC lung metastasis. Furthermore, immunohistochemistry results also revealed the increased expression of FN and vascular endothelial cell markers CD31 in lung tissues of the high expression level of Cav-1 group. Since they are a hallmark of ECM deposition and angiogenesis, these results further confirmed the conclusion. Altogether, our study illustrated that BC-derived exosomes could transport Cav-1 and promote the BC lung metastasis, providing a new perspective for studying the function of Cav-1 and BC lung metastasis.

When BC metastasizes, it has a tendency to move to specific sites such as the bone, lung, liver and brain 29, 30. This organotropism in BC leads to the discovery of metastatic microenvironments at these sites. The metastatic microenvironments are composed of unique resident cell types, ECM components, and infiltrating cell populations. All of the diverse cell types and complex cellular interactions have led to the concept of the metastatic niche 31. In our study, we have found that epithelial cells, fibroblasts and macrophages play an important role in PMN formation. One of the foundations and the earliest changes that define the PMN is the ECM deposition and remodeling 32. Researches have showed that secreted factors released from the primary tumor can change the expression of ECM proteins such as FN and TnC, which enhances the ability of BC cells to colonize the lung and form overt lung metastases 33-35. The presence of these matricellular proteins in metastatic lesions indicates the important roles of PMN in metastatic process 34. Our results found the expression of ECM components proteins emilin1, nidogen, TnC and FN increased in the lung fibroblasts after the cells were treated with BC-derived exosomes with the high expression level of Cav-1. Furthermore, we found TnC is a key component promoting exosome dependent tumor invasion. Studies have shown that TnC matrix deposition in Cav1-expressing fibroblasts depends on the biogenesis of exosome secretion 6. TnC can only be detected in the exosomes of MDA-MB-231 cells containing Cav-1 36. In our study, when TnC gene was knocked out in fibroblasts, the expression level of TnC in fibroblasts stimulated by BC-derived exosomes containing decreased Cav-1 was low, while the expression level of TnC in cells with increased Cav-1 was high. This suggested that Cav-1 in BC-derived exosomes can mediate cellular communication as a signaling molecule and regulate ECM deposition by regulating the expression level of TnC in lung fibroblasts.

In addition, during BC lung metastasis, lung epithelial cells also critical functions. Our study showed that the expression level of PMN marker genes CCL2, S100a8 in lung epithelial cells was increased after the stimulation of BC-derived exosomes containing increased Cav-1. The recruitment of CCL2 during PMN formation plays critical roles in cancer metastasis target organs. For example, CCL2 mediates lung overexpression of endogenous Toll-like receptor 4 (TLR4) ligands such as S100A8, which can improve the survival rate of cancer cells in the target organs 37. Endogenous TLR4 dependent innate immune system plays a critical role in lung PMN. Fibroblasts in the blood can recruit MDSCs by secreting CCL2, which also promotes the establishment of lung PMN and lung metastasis of melanoma cells 38. Because CCL2 is recruited in Gr1-positive inflammatory monocytes in the lung, the CCL2/CCR2 axis is also responsible for organ-specific metastasis of breast cancer 39. Altogether, these data suggest another possible mechanism that induces lung metastasis of BC by promoting the release of various cytokines and chemokines by lung epithelial cells to form a chronic inflammatory environment, thereby promoting lung metastasis of BC.

Macrophages are broadly classified as tumor suppressor M1 type and tumor promoter M2 type 40. M1 macrophages are usually recognized by the surface markers CD86 and CD64 41, 42, M2 macrophages usually express the surface markers CD206 and CD163 43. Our flow cytometry results showed that Cav-1 in BC-derived exosomes promoted the M2 polarization of macrophages. Studies have revealed that recruitment of monocytes/macrophages may be critical for metastatic cell survival and PMN establishment 16. Localization of monocytes to the site of inflammation mostly depends on the CCL2/CCR2 pair 44. Li, et al. showed that upon deletion of PTEN in macrophages, cytokines such as CCL2 that were known to promote macrophage polarization were overexpressed. There is enhanced recruitment of M2 macrophages, which results in increased angiogenesis through upregulation of VEGF-A as well as immune suppression in the tumor stroma 7. Similar results were observed in our cell models, we have found that the high expression level of Cav-1 decreased the expression of PTEN and increased the expression of CCL2 and VEGF-A, which indicated that the inhibition of the PTEN/CCL2/VEGF-A signaling pathway induced by Cav-1 in BC-derived exosomes may promote the M2 polarization of macrophages and facilitate the lung PMN formation.

## Conclusion

In summary, we found that exosomes play a role in transporting Cav-1 between in situ BC and the microenvironment of metastatic organs. Through upregulating the PMN-related genes in lung epithelial cells, regulating lung fibroblasts to induce ECM deposition, and inhibiting the PTEN/CCL2/VEGF-A signaling pathway of lung macrophages to affect M2-type polarization and angiogenesis, Cav-I in BC-derived exosomes promotes PMN formation and BC lung metastasis.

## Supplementary Material

Supplementary table.

## Abbreviations

Cav-1: Caveolin-1
TnC: tenascin-C
ECM: extracellular matrix
PMN: pre-metastatic niche
CCL2: Chemokine ligand 2
VEGF-A: Vascular endothelial growth factor A
BC: breast cancer
Cav-1: Caveolin-1
TnC: tenascin-C
ECM: extracellular matrix
PMN: pre-metastatic niche
CCL2: Chemokine ligand 2
VEGF-A: Vascular endothelial growth factor A
VEGF-B: Vascular endothelial growth factor B
MVB: multivesicular bodies
EMT: epithelial-mesenchymal transition
TSG: tumor susceptibility gene
CAF: cancer-associated fibroblasts
