# Supplementary material for: Tumor-derived Cav-1 promotes pre-metastatic niche formation and lung metastasis in breast cancer
Source: Theranostics. 2023 Mar 13;13(5):1684–97. doi: 10.7150/thno.79250 (PMC10086203; doi:10.7150/thno.79250)
Supplement: Supplementary file 1 — Supplementary table. [file thnov13p1684s1.pdf]

Supplemental Material

RT-qPCR primers were synthesized by Shanghai Shenggong Technology. The sequences of primers are shown in the table.

Gene	Primer	Sequence（5’-3’）
GAPDH	Forward	TCATCATCTCTGCCCCCTCT
	Reverse	AGTGATGGCATGGACTGTGG
Tenascin C	Forward	TTCAGTCGGAAACTGCCCTC
	Reverse	AGGAGGTGGTATCTGGACCC
Fibronectin	Forward	GCTCAAGTGGTCCTGTCGAA
	Reverse	TGAGATGGCTGTGGTGCATT
Nidogen	Forward	GGCTGTCAGTCAAAGCAAGC
	Reverse	TCGCAGTTAAAACCTCGGCT
Emilin	Forward	CCTGCGTCTTCCACACCAC
	Reverse	CTCTGCACCTGTTCCTCCAG
Cav-1	Forward	GCGACCCTAAACACCTCAAC
	Reverse	ATGCCGTGTCAAACTGTGTGTC
CCL2	Forward	TGAAGCTCGCACTCTCG
	Reverse	GTGACTGGGGCATTGATT
S1008	Forward	TGCTAGAGACCGAGTGTCCT
	Reverse	TGCCACGCCCATCTTTATCA
PTEN	Forward	GAGGGCCAGGTCATAAATAA
	Reverse	ACCATAAAATGTAAGCAAGGC
VEGF-A	Forward	ATTGGAGCCTTGCCTTG
	Reverse	CTCGATTGGATGGCAGTAG
VEGF-B	Forward	TATACTCGCGCTACCTGCCA
	Reverse	ACACTCCAGGCCATCGTCA
